# Association of spirochetal infection with Morgellons disease

**DOI:** 10.12688/f1000research.2-25.v1

**Published:** 2013-01-28

**Authors:** Marianne J Middelveen, Divya Burugu, Akhila Poruri, Jennie Burke, Peter J Mayne, Eva Sapi, Douglas G Kahn, Raphael B Stricker

**Affiliations:** 1 International Lyme and Associated Diseases Society, Bethesda, MD, 20827, USA; 2 Department of Biology and Environmental Science, University of New Haven, West Haven, CT, 06516, USA; 3 Australian Biologics, Sydney, 2000, Australia; 4 Department of Pathology, Olive View-UCLA Medical Center, Sylmar, CA, 91342, USA

## Abstract

Morgellons disease (MD) is an emerging multisystem illness characterized by skin lesions with unusual filaments embedded in or projecting from epithelial tissue. Filament formation results from abnormal keratin and collagen expression by epithelial-based keratinocytes and fibroblasts. Recent research comparing MD to bovine digital dermatitis, an animal infectious disease with similar skin features, provided clues that spirochetal infection could play an important role in the human disease as it does in the animal illness. Based on histological staining, immunofluorescent staining, electron microscopic imaging and polymerase chain reaction, we report the detection of *Borrelia *spirochetes in dermatological tissue of  four randomly-selected MD patients. The association of MD with spirochetal infection provides evidence that this infection may be a significant factor in the illness and refutes claims that MD lesions are self-inflicted and that people suffering from this disorder are delusional. Molecular characterization of the *Borrelia* spirochetes found in MD patients is warranted.

## Introduction

Morgellons disease (MD) is an evolving skin disease associated with filaments found beneath unbroken skin or projecting from spontaneously-appearing, slowly-healing skin lesions ^1^. In addition to dermopathy, patients may also exhibit debilitating musculoskeletal and neurological manifestations resembling the symptoms of Lyme disease ^1, 2^. Similarities were found between MD and bovine digital dermatitis (BDD), a disease common in dairy herds and characterized by keratin filament formation in skin lesions that frequently occur above the hind feet of cows ^3, 4^. Chronic BDD lesions demonstrate proliferation of long keratin filaments, and microscopic examination of histological sections from this tissue has revealed the presence of various *Treponema* spp. among enlarged keratinocytes throughout the stratum spinosum and dermal papillae ^5– 9^.

The etiology of BDD is considered to be multifactorial with coinvolvement of spirochetes and other bacterial pathogens ^10– 14^. In the animal disease, repeated detection of spirochetes from lesions and sero-reactivity to *Borrelia burgdorferi* antigens provides evidence of spirochetal involvement ^10– 14^. Successful experimental infection with tissue homogenates and pure cultured treponemes has confirmed that spirochetes are primary etiologic agents ^15, 16^.

Like BDD, MD filaments are produced by epithelial cells and stem from the stratum basale and from the root sheath of hair follicles, thus providing evidence that the filaments are cellular in origin ^3, 4^. Furthermore, immunohistochemical and histological staining has demonstrated that these filaments have a collagen as well as a keratin component ^5, 17^. Like cattle with BDD, patients with MD also produce antibodies reactive to *Borrelia burgdorferi* antigens ^18^. Multisystemic symptoms resembling Lyme disease also imply a possible spirochetal etiology for MD ^1– 3, 18, 19^. The frequent clinical diagnosis of Lyme disease and coinfecting tick-borne pathogens in MD patients suggests a multifactorial etiology and possible vectoring by ticks ^1– 3, 18, 19^.

In light of the proven spirochetal association with BDD and the possible association with MD, we undertook a histological, electron microscopic and PCR study of MD dermatological tissue samples to investigate the presence of spirochetes in these samples. In addition, bacterial culture was conducted to investigate the possibility of viable spirochetes in MD tissue.

## Materials and methods

### Patient selection and dermatological samples

Representative non-biopsy dermatological specimens were collected from four randomly-selected patients who met the key clinical criterion for MD, namely that filaments visible with a hand-held microscope at 60X magnification must be present under unbroken skin or projecting from spontaneously appearing skin lesions. Patients 1 and 2 are Americans residing in Texas while patients 3 and 4 are Canadians residing in Alberta, Canada ( Table 1). Written informed consent for submission of clinical samples and publication of clinical details and clinical images was obtained from each study subject. Patient anonymity and confidentiality were strictly maintained. The study was exempt from Institutional Review Board approval because all testing was performed as part of routine clinical care, and patient anonymity and confidentiality were strictly maintained.

**Table 1.** Summary of Morgellons disease patient data.

Patient	Age/Sex	Residence	RPR	Lyme serology	Delusional illness	Antibiotic therapy	Coinfections
1	72F	San Antonio, TX, USA	Negative	Positive	None	Currently taking doxycycline	Babesiosis and Bartonellosis
2	49F	Hughes Springs, TX, USA	Negative	Positive	None	Previous doxycycline therapy	Ehrlichiosis
3	54F	Cardston, AB, Canada	Negative	Positive	None	None	Unknown
4	73F	Calgary, AB, Canada	Negative	Positive	None	None	Unknown

RPR, rapid plasma reagin test.

The detailed histopathological findings in these patients were reported previously ^17^. All patients were seroreactive to *Borrelia burgdorferi* antigens (strains B31 and 297, IGeneX Laboratory, Palo Alto, CA) and negative on rapid plasma reagin (RPR) testing (RPR Card Test Kit, BD Diagnostic Systems, Sparks, MD). Patient 1 was on doxycycline therapy for Lyme disease at the time of the study, while patient 2 had previously been treated with doxycycline for Lyme disease but had been off treatment for several years at the time of the study. Patients 3 and 4 were not on antibiotic therapy at the time of the study. None of the study patients had evidence of a delusional disorder, as determined by standard neuropsychiatric testing using the Rorschach, Minnesota Multiphasic Personality Inventory (MMPI), Millon Clinical Multiaxial Inventory (MCMI) and Wechsler Adult Intelligence Scale (WAIS) formats.

The late-stage BDD biopsies used for comparison were kindly provided by Dr. Dorte Döpfer, Faculty of Veterinary Medicine, University of Wisconsin, Madison, WI. Biopsies were taken as part of an intervention study conducted by the University of Wisconsin ^15^. The diagnostic criterion for late-stage BDD was the presence of pronounced keratin projections from ulcerative lesions that were at least two centimeters in diameter and located above the heel bulb of the hind feet of cattle. Biopsy samples were stored and shipped in a fixative of 1.5% glutaraldehyde/1.0% formaldehyde in Sorensen’s Buffer at pH 7.35 (Tousimis Research Corporation, Rockville, MD). Duplicate samples were used for each of the light and electron microscopic studies described below.

### Light microscopy

The gross morphology of dermatological specimens collected from Patients 1–4 was observed at 8X, 40X, and 100X magnification with illumination superior to the specimen, thus verifying the presence of filaments within and protruding from epithelial tissue. BDD biopsy material was examined at 8X to observe gross morphological characteristics.

Morgellons samples were formalin-fixed and embedded in paraffin, sectioned, and stained with Warthin-Starry and/or Dieterle silver nitrate-based staining for the light microscopic detection of spirochetes under oil immersion at 1000X magnification. Warthin-Starry staining and Dieterle staining were performed by Interscope Pathology Medical Group, Canoga Park, CA, and McClain Laboratories LLC, Smithtown, NY, respectively.

BDD biopsies were formalin-fixed and embedded in paraffin, sectioned, and stained for the detection of spirochetes by Warthin-Faulkner silver nitrate-based staining at Prairie Diagnostics, University of Saskatchewan, Saskatoon, Saskatchewan.

Formalin-fixed paraffin-embedded MD sections were processed for immunofluorescent anti- *Borrelia* staining and imaging as previously described ^20^ at the University of New Haven, West Haven, CT, by the following protocol: fixed specimens were pre-incubated with 10% normal goat serum (Thermo Fisher Scientific, Waltham, MA) in PBS containing 0.5% bovine serum albumin (BSA) (Sigma-Aldrich, St. Louis, MO) for 30 minutes to block non-specific binding of the secondary antibody. The slides were washed with PBS containing 0.5% BSA and then incubated for 1 hour with fluorescein isothiocyanate (FITC)-labelled *Borrelia*-specific polyclonal antibody (Thermo Fisher Scientific, #73005) at a 1:50 dilution in PBS containing 1% BSA pH 7.4. The slides were washed and then counterstained with 4’, 6-diamidino-2-phenylindole (DAPI) for 10 minutes. In negative control samples, anti-specifically targeted antibody was replaced with normal rabbit IgG (Vector Laboratories, Burlingame, CA, #I-1000). Mounted slides were imaged using fluorescent microscopy.

### Electron microscopy

Morgellons and BDD samples were fixed in buffered 2.5% glutaraldehyde. Scanning electron microscopy (SEM) and transmission electron microscopy (TEM) were performed by the Electron Microscopy Facility, Department of Materials Science and Engineering, Clemson University, Anderson, SC, according to the protocols below:

***SEM.*** Glutaraldehyde-fixed samples for SEM were washed in buffer and dehydrated in a graded series of ethanol concentrations. Samples were then immersed in hexamethyldisilazane (Electron Microscopy Sciences, Hatfield, PA) for 5–15 minutes and air dried at room temperature. Dried samples were mounted on A-1 mounts. Samples were not coated but placed into a Hitachi TM3000 microscope and imaged in the variable pressure mode.

***TEM.*** Glutaraldehyde-fixed samples were washed in buffer, followed by dehydration in a graded series of ethanol concentrations. Samples were then immersed in a 50:50 mixture of LR White™ embedding resin and 100% ethanol for 30 minutes, followed by pure LR White™ resin until samples settled on the bottom of the vial. The resin-immersed samples were then placed into pure resin in beam capsules and put into a 60°C oven overnight for polymerization. Sections were cut on an Ultracut E microtome to produce sections 60–90 nm thick, placed onto copper grids and stained in uranyl acetate for 20 minutes. Images were taken on a Hitachi 7600 microscope.

### PCR

Morgellons calluses from patients 1–4 were forwarded to Australian Biologics (Sydney, Australia) for *B. burgdorferi* detection by PCR using the Eco™ Real-Time PCR system with software version 3.0.16.0. DNA was extracted from the tissue samples using the QIAamp DNA Mini Kit (QIAGEN). The four samples were analyzed in duplicate with positive and negative controls using primers AB-B1 for the *Borrelia* 16S rRNA gene target, as previously described ^21^. The thermal profile for all analyses involved incubation for 2 mins at 50°C, polymerase activation for 10 mins at 95°C then PCR cycling for 40 cycles of 10 secs at 95°C dropping to 60°C sustained for 45 secs.

The magnitude of the PCR signal generated (∆R) for each sample was interpreted as positive or negative compared to positive and negative controls.

### *Borrelia* spp. culture

Borrelial culture was performed as described previously ^22^. *B. burgdorferi* was cultured in Barbour–Stoner–Kelly H (BSK-H) complete medium, with 6% rabbit serum (Sigma Aldrich, #B8291) and the following antibiotics: phosphomycin (0.02 mg/l), rifampicin (0.05 mg/l), and amphotericin B (2.5 µg/l) (Sigma-Aldrich) and incubated at 32°C with 5% CO _2_. Cultured spirochetes were observed by dark-field microscopy and/or heat-fixed and stained with crystal violet (Dalynn Biologicals, Calgary, AB) under oil immersion at 1000X. For the immunofluorescence studies, cultured spirochetes (1×10 ^7^ individual spirochete cells) were centrifuged at 8,000×g for 10 minutes at room temperature, washed once with PBS pH 7.4, and then centrifuged again at 8,000×g for 10 minutes at room temperature. The pellet was resuspended in 100 µl of PBS pH 7.4, and then spread on microscope slides (SuperFrost+, Thermo Fisher Scientific). Spirochetes were fixed by incubating the slides in cold acetone for 10 minutes at -20ºC. Slides were then washed twice with PBS pH 7.4 at room temperature, and immunofluorescent staining with polyclonal anti- *Borrelia* antibodies was performed as described above.

## Results

### Gross microscopic observations

**Figure 1.**
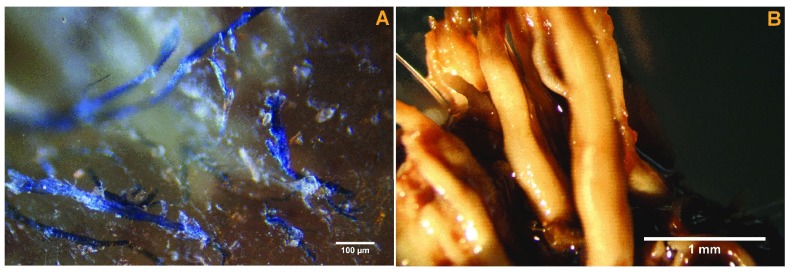
**A**) Morgellons disease filaments embedded in and projecting from epithelial tissue, 100X magnification. **B**) Proliferative bovine digital dermatitis (BDD) keratin filaments, 8x magnification.

Calluses from the four MD patients demonstrated white, red and blue filaments, alone or in any color combination, each 10 to 40 µm in diameter, embedded in or projecting from epithelial tissue ( Figure 1A). BDD biopsies demonstrated pronounced, unusual keratin filament production typical of late-stage proliferative infection ( Figure 1B).

A summary of the following histological, culture, electron microscopic and PCR results is shown in Table 2.

**Table 2.** Summary of laboratory findings in Morgellons disease patients.

Patient	Silver nitrate staining	Culture	IFA staining	SEM/TEM	PCR
1	Spirochetes detected	Not performed	Positive, histological sections	Spirochetes observed, TEM	Weak positive
2	Spirochetes detected	Not performed	Positive, histological sections	Spirochetes observed, both SEM and TEM	Positive
3	Spirochetes detected	Positive, motile spirochetes detected, confirmed by IFA staining	Positive, both histological sections and cultured spirochetes	Not performed	Positive
4	Spirochetes detected	Positive, motile spirochetes detected	Positive, histological sections	Not performed	Negative

IFA, immunofluorescence assay; SEM, scanning electron microscopy; TEM, transmission electron microscopy; PCR, polymerase chain reaction.

### Light microscopy

**Figure 2.**
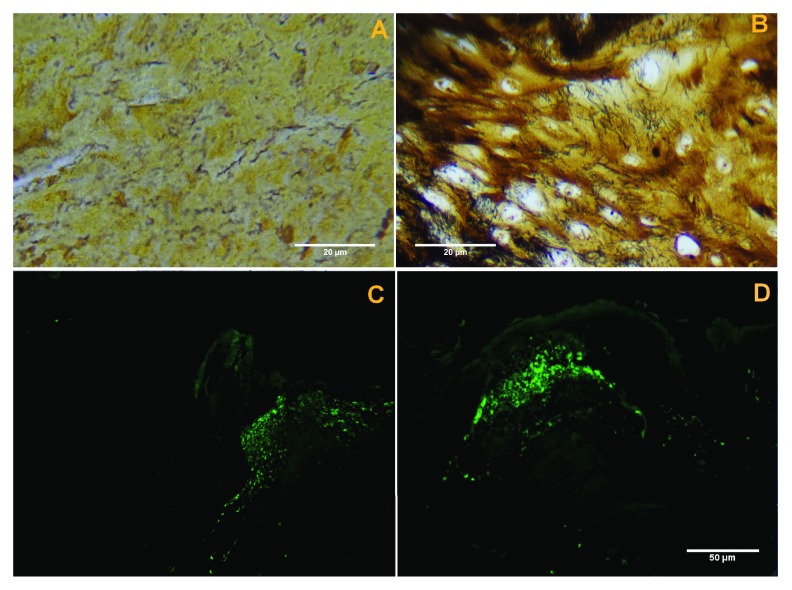
**A**) Black-stained spirochetes in representative tissue sample from patient 2. Dieterle stain, 1000X oil immersion. **B**) Black-stained spirochetes in bovine digital dermatitis (BDD) tissue sample, Warthin-Faulkner stain, 1000X oil immersion. **C**) Distinct patches of anti- *Borrelia* fluorescence in histological section of callus from patient 1, 400X magnification. **D**) Distinct patches of anti- *Borrelia* fluorescence in histological section of callus from patient 2, 400X magnification.

***Silver nitrate-based staining.*** Staining of dermatological tissue from patients 1–4 revealed visible black-stained spirochetes among keratinocytes and inflammatory cells ( Figure 2A). These spiral or curved structures ranged from 0.1 µm to 0.5 µm in diameter and up to 30 µm long, and they were present mostly in the interior areas of the sections and not along the peripheral edge.

Staining of BDD dermatological tissue revealed visible black-stained spirochetes among enlarged keratinocytes ( Figure 2B). Spirochetes were approximately 0.1 µm to 0.2 µm in diameter and approximately 10 µm to 15 µm in length, and they varied in morphology from visibly spiral-shaped to straight or wavy in appearence.

***Immunofluorescent anti-Borrelia staining.*** Immunofluorescent anti- *Borrelia* staining of fixed *Treponema denticola* spirochetes was performed as a negative control and immunofluorescence was not observed for these specimens (data not shown). Histological sections of dermatological material from patients 1–4 all demonstrated distinct patches of immunofluorescence at a magnification of 400X ( Figures 2C and 2D). Patches of fluorescence appeared to occur most often in areas of sections corresponding to fibroblasts. Cultured spirochetes from patient 3 also demonstrated positive immunofluorescent staining with anti- *Borrelia* antibodies (see below and Figure 5C).

### SEM and TEM

**Figure 3.**
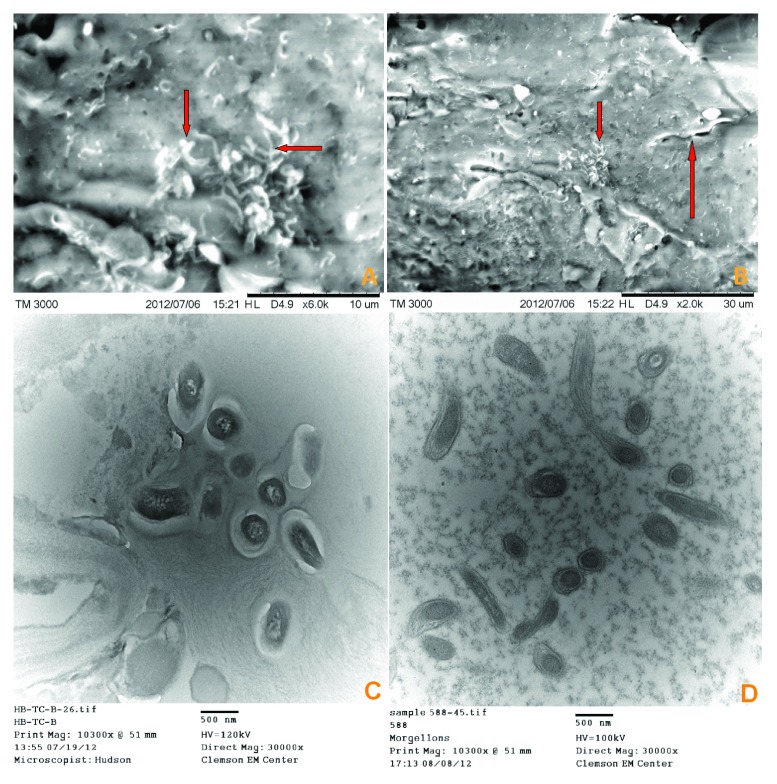
**A**) SEM from patient 2 tissue sample showing spirochete images that are consistent with morphological forms of *Borrelia* (arrows). **B**) SEM from patient 2 tissue sample showing single spirochete, upper middle right (long arrow) and morphological forms consistent with *Borrelia*, center (short arrow). **C**) TEM from patient 1 tissue sample showing sectioned spirochetes. **D**) TEM from BDD tissue sample showing sectioned spirochetes.

SEM revealed high-resolution surface imaging of a spirochete lying beneath a layer of dermatological tissue of a Morgellons callus and images consistent with morphological forms of *Borrelia* spp. ( Figures 3A and 3B). TEM imaging of both Morgellons calluses and BDD biopsies revealed spirochetes in cross-section ( Figures 3C and 3D).

### PCR

**Figure 4.**
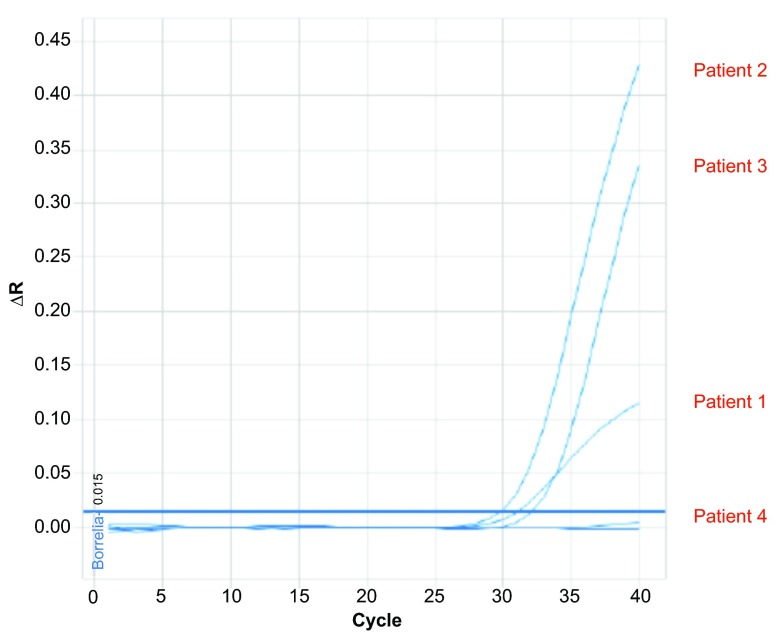
Results of polymerase chain reaction (PCR) amplification in tissue samples from patients 1–4. See text for PCR protocol. Samples from patients 2 and 3 were strongly positive, while the sample from patient 1 was weakly positive. The sample from patient 4 was negative.

Real-time PCR analysis was positive for Borrelial DNA in tissue samples from MD patients 1–3 and negative in the sample from Patient 4. Samples from Patients 2 and 3 were clear positives and the sample from Patient 1 was a weak positive. The PCR profiles are shown in Figure 4.

### *Borrelia* spp. culture

**Figure 5.**
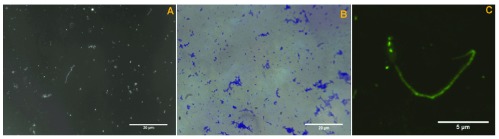
**A**) Cultured spirochetes from patient 3 tissue samples, 1000X darkfield microscopy, oil immersion. **B**) Cultured spirochetes in clumps from patient 4 tissue sample, heat fixed and stained with crystal violet, 1000X oil immersion. **C**) Borrelial spirochetes demonstrating fluorescence from tissue culture of patient 3, 1000X oil immersion, enlarged and cropped.

Motile spirochetes ranging from approximately 0.1 µm to 0.5 µm in diameter and up to 30 µm long were visible in cultures inoculated with dermatological tissue from both patients 3 and 4 ( Figures 5A and 5B). Cultured spirochetes from patient 3 were identified as *Borrelia* by immunofluorescent staining with FITC-labelled polyclonal antibodies at 1000X magnification ( Figure 5C). The culture obtained from the inoculum from patient 4 was lost due to contamination and generic identification was not obtained.

## Discussion

The presence of spirochetes in MD dermatological specimens demonstrates that Morgellons lesions are associated with spirochetal infection. Unlike *Treponema pallidum* spirochetes, which are seldom detected in secondary and tertiary syphilitic skin lesions ^23– 25^, spirochetes were readily detectable in dermatological tissue from four MD patients using a combination of immunohistochemical, electron microscopic and PCR techniques. Motile spirochetes were also observed in cultures inoculated with MD dermatological tissue, thus indicating that our specimens contained viable organisms. These findings are similar to the observation of significant spirochetal loads in lesions of cattle with BDD, suggesting that spirochetes could be associated with unusual filament production in both bovines and humans. Although Ekbom reported that syphilitic infection was associated with feelings of infestation ^26^, to our knowledge dermal fibers have not been reported in patients with syphilis.

Unlike BDD, which is associated with a variety of treponemal spirochetes ^15, 16^, the MD dermatological tissue in this study contained spirochetes that were identified as *Borrelia* by immunofluorescent staining with anti- *Borrelia* antibodies. Furthermore the MD spirochetes were specifically classified by targeted PCR as *Borrelia burgdorferi*. Given the fact that all four MD patients in this study were seroreactive to *Borrelia burgdorferi* antigens, some of which are thought to be species-specific, and were RPR negative, we speculate that the Morgellons phenomenon observed in our group of study patients is a manifestation of Lyme disease. At present it is not understood if MD filaments are associated exclusively with *Borrelia burgdorferi sensu stricto*, perhaps a particular genotype, or with a *Borrelia* species more appropriately placed in the *Borrelia burgdorferi sensu lato* complex. As our study sample was small, we cannot ascertain at this stage whether Morgellons filaments are associated with spirochetes belonging to other genera as well as *Borrelia*.

The etiology of MD appears to be multifactorial, and at this stage secondary etiologic factors are not well understood. MD is most often reported in middle-aged Caucasian females. It is a disease reported mostly in the Northern Hemisphere, and it is often associated with known tick exposure, a Lyme disease diagnosis, and serological evidence of coinfecting tick-borne agents ^1, 2, 18, 19^. Two of our study patients had laboratory-confirmed tick-borne coinfections (see Table 1), and these coinfections may contribute to the pathology of this disease.

The filaments seen in MD are composed of keratin and collagen derived from keratinocytes and fibroblasts, respectively ^4, 17^. We hypothesize that spirochetes associated with MD trigger the production of unusual collagen and keratin filaments. In our study, spirochetes were detected in Morgellons dermatological tissue from both Patient 1, who was currently taking antibiotics, and from Patient 2, who had been on antibiotic therapy in the past but was not on treatment at the time that samples were obtained. *B. burgdorferi* has been reported to invade human fibroblasts, and viable *B. burgdoferi* spirochetes have been isolated from lysates of fibroblast monolayers, even after antibiotic therapy ^27^. Our findings suggest that *Borrelia* spirochetes may be capable of sequestering within keratinocytes and fibroblasts, causing both persistent infection that is refractory to antibiotic therapy and aberrant fiber production by these infected cells in MD patients.

Despite contrary evidence, some medical professionals have attributed MD to delusions of parasitosis or delusional infestation. MD is thought to result from psychiatric illness and is diagnosed on the basis of patient belief in infestation by parasites, or the presence of inanimate objects such as fibers that are thought to be deliberately self-implanted ^28– 32^. As stated above, spirochetal infection associated with itching and crawling sensations and feelings of infestation dates as far back as 1945 in Ekbom’s original description of delusions of parasitosis ^26^, and many of the patients in that study were diagnosed with syphilis. This clinical observation provides valuable insight into MD.

The insistence that MD is delusional has prevented the establishment of universally accepted, objective diagnostic criteria for this disease. Consequently, some studies have included diverse groups of research subjects, including patients who may not actually have had MD ^30– 32^. In the present study, the key diagnostic criterion is that filaments visible with a hand-held microscope at 60X magnification must be present under unbroken skin or projecting from spontaneously-appearing skin lesions ^1, 2^. This important clinical feature forms the basis for an accurate MD diagnosis.

Although a study from the Centers for Disease Control and Prevention (CDC) found no evidence that pathogens play a role in MD, the search for spirochetal pathogens in that study was confined to Warthin-Starry staining on limited tissue samples and commercial serological testing for *Borrelia burgdorferi* ^31^. Tissue staining in that study was performed on samples from patients who reportedly did not have confirmed clinical evidence of MD ^32^, and serological testing was interpreted in accordance with Lyme surveillance criteria that are inappropriate for clinical diagnosis ^33^. Thus the findings in the CDC study were influenced by failure to examine the appropriate group of patients and by the clinical insensitivity of surveillance testing for tickborne disease ^32, 33^. These limitations leave open the possibility that a spirochetal association with MD could have been missed in the CDC study.

Controversy surrounding MD has been detrimental to those afflicted with this illness. It has stifled scientific research and has prevented appropriate treatment and control strategies from being investigated and implemented. In some cases it has resulted in treatment with ineffective and potentially harmful antipsychotic drugs ^28– 32^. Some patients have been stigmatized by a diagnosis of mental illness that resulted in social isolation, loss of employment, loss of custody of children, and a high rate of suicide (Casey C. 2012. Personal communication. http://www.thecehf.org/). Further MD research is urgently needed to delineate the possible infectious etiology of the disease and to assure that patients can be appropriately diagnosed and treated in the future.

## Conclusions

This report demonstrates the presence of *Borrelia* spirochetes in dermatological samples collected from four MD patients who were seroreactive to *Borrelia burgdorferi* antigens. The findings suggest that MD has a spirochetal etiology and raises the possibility that this emerging dermopathy may be a manifestation of Lyme disease in a subgroup of tickborne disease patients. The demonstration of an infectious agent associated with MD contradicts the belief that patients with this disease suffer from a factitious or delusional illness. Although our sample size was small, our study indicates that, at least in some patients, MD appears to be an important emerging infectious disease. Further research is needed to assure the correct diagnosis and define the optimal treatment for this spirochetal infection so that MD patients are not stigmatized with a diagnosis of mental illness.
