# Supplementary material for: Association of spirochetal infection with Morgellons disease
Source: F1000Res. 2013 Jan 28;2:25. [Version 1] doi: 10.12688/f1000research.2-25.v1 (PMC3954163; doi:10.12688/f1000research.2-25.v1)
Supplement: Peer review history

# Peer review: Referee response for version 1

**Published:** 2013-04-03

**Authors:** John English

**Affiliations:** 1 Department of Dermatology, Queen's Medical Centre, Nottingham University Hospitals NHS Trust, Nottingham, UK

It would be nice to know if treating the *Borrelia *helped the Morgellons disease?

**Publisher’s note added on 19 Apr 2013: **Since the publication of this referee report, a potential conflict of interest has come to light. It appears that just prior to John English completing his report, he and Peter Mayne had been collaborating on another manuscript. Following the publication of the article by Middelveen *et al.* in *F1000Research*, Peter Mayne asked Raphael Stricker to become an additional author on this subsequent manuscript, which has since been submitted to *F1000Research* with Stricker, Mayne and English among the authors. Given this potential conflict of interest, the original referee status has been greyed out on this report but the comments of the reviewer on the article are still accessible.

I have read this submission. I believe that I have an appropriate level of expertise to confirm that it is of an acceptable scientific standard.

# Peer review: Referee response for version 1

**Published:** 2013-04-03

**Authors:** Bernhard Zelger

**Affiliations:** 1 Department of Dermatology, Medical University Innsbruck, Innsbruck, Austria

I have read this submission. I believe that I have an appropriate level of expertise to confirm that it is of an acceptable scientific standard.

# Peer review: Referee response for version 1

**Published:** 2013-04-03

**Authors:** Judith Miklossy

**Affiliations:** 1 International Alzheimer Research Center, Alzheimer Prevention Foundation, Martigny-Croix, Switzerland

Pathological characteristics of skin lesions in bovine digital dermatitis and Morgellons disease (MD) show strong similarities. As the role of spirochetal infection in bovine digital dermatitis is established in a comparative analysis, the authors examined the presence of spirochetes in Morgellons disease. They detected *Borrelia* spirochetes in skin lesions in four randomly selected Morgellons patients who were seroreactive to *Borrelia burgdorferi*. For the detection of spirochetes various techniques were used, including histochemistry, immunohistochemistry, electron microscopy and polymerase chain reaction. They conclude that spirochetal infection may be a significant factor in the illness and refute claims that the symptoms of patients are delusional and that Morgellons skin lesions are self-inflicted.

This is an important work showing invasion of skin lesions by spirochetes in four Morgellons patients with positive *Borrelia* serology. The manuscript is well written, and importantly, the authors used various techniques for the detection of spirochetes, which is crucial for such a study. Their results are convincing and only a few and minor comments or changes are suggested, and some propositions for future research. I share the opinion of the authors that these skin lesions are not self-inflicted and they do not correspond to delusional parasitosis. Infection is a significant factor in their pathogenesis. 

1. “Representative non-biopsy dermatological specimens” or “representative tissue sample” might be better defined: In the Material and Methods it might be useful for those who would like to perform similar studies to indicate the site of tissue samples, their size and also how they were taken and how they were stored until the analysis. 

2. With respect to the detailed histopathological findings of these patients, the authors refer to their previous report (Middelveen *et al*., 2013) but an inflammatory component of skin lesions cannot be found, probably as skin biopsy was not performed. In future studies, the presence of an inflammatory reaction might be important to analyze as it will support an infectious origin of skin lesions. 

3. Figures and figure legends

Figure 1: B) With respect to the keratin filaments of bovine digital dermatitis (BDD), it would be useful to insert the method used or refer to a previous publication. 

Figure 2: Representative tissue sample – should be more precise. It is not clear whether panel A is a histological section or not? Paraffin or frozen section from skin lesions?

Figure 3: Panels A and B were both taken from the same region of a sample from patient 2. The presence of spirochetes is convincing, however the large “spirochete” in panel B is not comparable to the group of spirochetes seen in the center. I would suggest removing panel B.

Figure 5 : Panel B: Heat shock instantly induces atypical and agglutinated forms of spirochetes, therefore, this panel is not representative to show cultured spirochetes. I would recommend removing this panel. The authors have enough other convincing results. It is not necessary for the study. 

C: Probably the spirochete was visualized by immunofluorescence using *Borrelia*-specific antibody. This should be mentioned.

4. " *Although a study from the Centers for Disease Control and Prevention (CDC) found no evidence that pathogens play a role in MD, the search for spirochetal pathogens in that study was confined to Warthin-Starry staining on limited tissue samples and commercial serological testing for Borrelia burgdorferi ^31^. Tissue staining in that study was performed on samples from patients who reportedly did not have confirmed clinical evidence of MD ^32^, and serological testing was interpreted in accordance with Lyme surveillance criteria that are inappropriate for clinical diagnosis ^33^. Thus the findings in the CDC study were influenced by failure to examine the appropriate group of patients and by the clinical insensitivity of surveillance testing for tickborne disease ^32,33^. These limitations leave open the possibility that a spirochetal association with MD could have been missed in the CDC study.*"

The work done by the “Unexplained Dermopathy Study Team” was an important initiative and a well organized and executed work (Pearson *et al*., 2012), however the population examined in the study was indeed not homogeneous enough, which can make the interpretation of the results difficult. In case definition, to consider the presence of the unusual blue, red or white filaments and if possible, the same stage of skin lesion, would be important. That the authors of this paper examined four Morgellons patients with a positive serology for *Borrelia burgdorferi* and the case definition of CDC study did not include positive serology for this spirochete may also explain why they did not find *Borrelia* in their cases. The discrepancy of the results may also be explained if, following the example of bovine digital dermatitis (Döpfer *et al*., 2012) we anticipate that various types of *Treponema* species might also be involved in MD. Perhaps the authors may wish to mention that further studies in this direction would be useful. Indeed spirochetes are present not only in ticks but in various other ectoparasites as well. Morphologically diverse populations of spirochetes are abundant and consistently present in termite hindguts (Paster *et al*., 1996; Ohkuma *et al*., 1999; Breznak, 2002). *Borrelia burgdorferi sensu lato* was also detected in mites (Netusil *et al*., 2005). Spirochetes were detected in lice and also in snails around lakes and fresh waters (Sitnikova *et al*., 2012). Both humans and cattle are at risk for infection.

5. “ *Filament formation results from abnormal keratin and collagen expression by epithelial-based keratinocytes and fibroblasts”; "Like BDD, MD filaments are produced by epithelial cells and stem from the stratum basale and from the root sheath of hair follicles, thus providing evidence that the filaments are cellular in origin. Furthermore, immunohistochemical and histological staining has demonstrated that these filaments have a collagen as well as a keratin component ^5,17^.*” 

I would suggest to stay open with respect to the origin of dark and white filaments and just refer to the literature “it was suggested” or “reported” (References) as we have not enough data for a definite conclusion. Helminths show similar properties to those of Morgellons filaments. They show morphological similarities, they are birefringent, they show autofluorescence, and contain keratin- (Shibui *et al*., 2001; Shinn *et al*., 1995; Cottee *et al*., 2006) and collagen-like proteins (e.g. Johnston, 1994; Yang *et al*., 2012; Kingston and Pettitt, 1990). In schistosomiasis the coexistence of blue and white adult worms is well known. Particularly, the adult female is frequently described as a dark or dark-blue elongated filament (Figure 1 in Kolárová *et al*., 2010), and cercarial forms (Figure 3 in Savely *et al*., 2006), might also occur in Morgellons disease. The skin manifestations of cercarial dermatitis or swimmer’s itch caused by bird schistosomes are also similar. Compare the skin manifestations in a Morgellons patient with a patient having cercarial dermatitis or swimmer’s itch. I would encourage research in this direction. Recent findings show that the parasite may migrate throughout the viscera and the nervous system of mammals, suggesting that the pathogenicity of *Trichobilharzia* may have been underestimated in the past (Horak *et al*., 2002). Schistosomiasis is one of the most prevalent parasitic infections worldwide. One should also consider that several helminths can infect together. 

6. Melanin pigments are produced by a broad variety of microorganisms, including bacteria, fungi, and helminths (Taborda *et al*., 2008, Esposito *et al.*, 2012) and a high amount of melanin accumulates in the host around invading worms and cercaria (Babu and Hall, 1974), In this view the Fontana-Masson-stained section from patient 3 from a previous work of the authors (see Figure 3 in Middelveen *et al*., 2013) demonstrating positive melanin staining “of blue filament sections (arrow)” might be compatible with helminthiasis. 

7. Epidermal parasitic skin diseases occur worldwide and a wide range of parasitic infections can involve the skin and subcutaneous tissues, including protozoa, trematodes, cestodes, nematodes and arthropods (Goldsmid and Melrose, 2005). Despite the considerable burden caused by them, they have been widely neglected by the scientific community and health-care providers (Feldmeier & Heukelbach, 2009). The WHO has included them in a program to remediate this situation. Following the various findings available with respect to 'Morgellons disease' it seems that various parasites should be considered and investigated in each case. Efficient treatments would be available. In order to contribute to this goal and to avoid controversies, open discussions will be necessary. Further research is essential.

I have read this submission. I believe that I have an appropriate level of expertise to confirm that it is of an acceptable scientific standard.

## References

[1] CogswellAAKommerVPWilliamsDL: Transcriptional analysis of a unique set of genes involved in Schistosoma mansoni female reproductive biology.*Plos One.*.2012;6(11) : 2510.1371/journal.pntd.000190723166854PMC3499410

[2] BabuJPHallJE: Histochemical Staining of Melanin Formed in a Mayfly in Response to Helminth Infection.*J. Intervertebrate Pathol.*.1974;123-124Reference source

[3] BreznakJA: Phylogenetic diversity and physiology of termite gut spirochetes.*Integr Comp Biol*.2002;42(2) : 25313-31810.1093/icb/42.2.31321708723

[4] CotteePANisbetAJAbs El-OstaYGWebsterTLGasserRB: Construction of gender-enriched cDNA archives for adult Oesophagostomum dentatum by suppressive-subtractive hybridization and a microarray analysis of expressed sequence tags.*Parasitology*.2006;132(Part 5) : 25691-70810.1017/S003118200500972816426483

[5] DöpferDAnklamKMikheilDet al.: Growth curves and morphology of three Treponema subtypes isolated from digital dermatitis in cattle.*Vet J.*.2012;193(3) : 25685-9310.1016/j.tvjl.2012.06.05422901455

[6] EspositoRD’AnielloSSquarzoniPPezzottiMRRistoratoreFSpagnuoloA: New Insights into the Evolution of Metazoan Tyrosinase Gene Family. *Plos One.*.2012;7(4) : 2510.1371/journal.pone.003573122536431PMC3334994

[7] FeldmeierHHeukelbackJ: Epidermal parasitic skin diseases: a neglected category of poverty-associated plagues.*Bulletin of the World Health Organization*.2009;87(2) : 25152-910.1590/S0042-9686200900020001819274368PMC2636197

[8] GoldsmidJMMelroseW: Parasitic infections of the skin. *Primer of Topical Medicine*. Reference source

[9] HorákPKolárováL: Biology of the schistosome genus Trichobilharzia.*Adv Parasitol.*.2002;52:155-2331252126110.1016/s0065-308x(02)52012-1

[10] JohnstoneIL: The cuticle of the nematode Caenorhabditis elegans: a complex collagen structure.*Bioessays.*.1994;16(3) :171-8816667010.1002/bies.950160307

[11] KingstonIBPettittJ: Structure and expression of Ascaris suum collagen genes: a comparison with Caenorhabditis elegans.*Acta Trop.*.1990;47(5-6) :280-7197852810.1016/0001-706x(90)90029-y

[12] KolárováLHorákPSkírnissonK: Methodical approaches in the identification of areas with a potential risk of infection by bird schistosomes causing cercarial dermatitis.*J. Helminthol*.2010;84(3) : 25327-3510.1017/S0022149X0999072120102677

[13] MiddelveenMJKahnDGStrickerRB: Characterization and evolution of dermal filaments from patients with Morgellons disease.*Clinical, Cosmetic and Investigational Dermatology.*.2013;6: 251-2110.2147/CCID.S3901723326202PMC3544355

[14] NetusilJZákovskáAHorváthRDendisMJanouskovcováE: Presence of Borrelia burgdorferi sensu lato in mites parasitizing small rodents.*Vector Borne Zoonotic Dis.*.2005;5: 25227-23210.1089/vbz.2005.5.22716187890

[15] OhkumaMLidaTKudoT: Phylogenetic relationships of symbiotic spirochetes in the gut of diverse termites.*FEMS Microbiol Lett.*.1999;181: 25123-12910.1016/S0378-1097(99)00526-110564797

[16] PasterBJDewhirstFECookeSMFussingVPoulsenLKBreznakJA: Phylogeny of not-yet-cultured spirochetes from termite guts.*Appl Environ Microbiol.*.1996;62:347-352Reference source859304010.1128/aem.62.2.347-352.1996PMC167805

[17] PearsonMLSelbyJVKatzKACantrellVBradenCRPariseMEPaddockCDLewin-SmithMRKalasinskyVFGoldsteinFCHightowerAWPapierALewisBMotiparaSEberhardMLUnexplained Dermopathy Study Team.: Clinical, epidemiologic, histopathologic and molecular features of an unexplained dermopathy.*PLoS One.*.2012;7: 25e2990810.1371/journal.pone.002990822295070PMC3266263

[18] SavelyVRLeitaoMMStrickerRB: The mystery of Morgellons: infection or delusion?. *Am J Clin Dermatol.*.2006;7:1-5Reference source1648983810.2165/00128071-200607010-00001

[19] ShibuiATakamotoMShiYKomiyamaASuganeK: Cloning and characterization of a novel gene encoding keratin-like protein from nematode Nippostrongylus brasiliensis.*Biochim Biophys Acta.*.2001;1522: 2559-6110.1016/S0167-4781(01)00300-111718901

[20] ShinnAPGibsonDISommervilleC: A study of the composition of the sclerites of Gyrodactylus Nordmann, 1832 (Monogenea) using X-ray elemental analysis.*Int J Parasitol.*.1995;25: 25797-80510.1016/0020-7519(95)00008-P7558565

[21] SitnikovaTMichelETulupovaYKhanaevIParfenovaVProzorovaL: Spirochetes in gastropods from Lake Baikal and North American freshwaters: new multi-family, multi-habitat host records.*Symbiosis.*.2012;56: 25103-11010.1007/s13199-012-0167-1

[22] TabordaCPda SilvaMBNosanchukJDTravassosLR: Melanin as a virulence factor of Paracoccidioides brasiliensis and other dimorphic pathogenic fungi: a minireview.*Mycopathologia.*.2008;165:331-3391877763710.1007/s11046-007-9061-4PMC2586806

[23] YangYJinYLiuPShiYCaoYLiuJShiYLiHLinJ: RNAi silencing of type V collagen in Schistosoma japonicum affects parasite morphology, spawning, and hatching.*Parasitol Res.*.2012;111: 251251-125710.1007/s00436-012-2959-x22638918
